# Further Description and First Genetic Characterization of *Oswaldofilaria bacillaris* (Nematoda: Onchocercidae) Infecting the Pantanal Caiman (*Caiman yacare*), with Insights into the Phylogeny of *Oswaldofilaria*

**DOI:** 10.1007/s11686-026-01264-7

**Published:** 2026-03-24

**Authors:** Glória M. C. Lacerda, Gustavo M. do Carmo, Lorena G. Ailán-Choke, Fernando Paiva, Luiz E. R. Tavares, João A. de Araújo-Filho, Samuel C. Ribeiro, Felipe B. Pereira

**Affiliations:** 1 Programa de Pós-Graduação em Parasitologia, Universidade Federal de Minas Gerais, Av. Antônio Carlos 6627, Pampulha, Belo Horizonte, MG 31270-901 Brazil; 2 Centro de Ecología Aplicada del Litoral, Consejo Nacional de Intestigaciones Científicas y Técnicas, Ruta Provincial 5, Km 2,5, Corrientes, CP. 3400 Argentina; 3 Instituto de Biociências, Universidade Federal de Mato Grosso do Sul, Av. Costa e Silva s/n, Campo Grande, MS 79070-900 Brazil; 4 Departamento de Ciências Biológicas, Centro de Ciências Biológicas e da Saúde, Universidade Regional do Cariri, Rua Vicente Alexandrino de Alencar, 348, Centro, Campos Sales, Crato, CE 63150-000 Brazil; 5 Laboratório de Biologia e Ecologia de Animais Silvestres—LABEAS, Instituto de Formação de Educadores, Universidade Federal do Cariri—UFCA, Rua Olegário Emidio de Araujo, s.n, Brejo Santo, Brazil

**Keywords:** Filarid, Oswaldofilariinae, Integrative taxonomy, Reptile, Crocodilia, Brazil

## Abstract

**Purpose:**

*Oswaldofilaria bacillaris* is the type species of the genus, but its morphology remains little known and partially contradictory. Moreover, there are no genetic data and scanning electron microscopical observations on the species, like in most *Oswaldofilaria* spp. The phylogenetic relationships among *Oswaldofilaria* spp. are practically unknown. This study aimed to evaluate the detailed morphology of *O*. *bacillaris*, provide its first genetic characterization, and discuss the phylogenetic relationships within *Oswaldofilaria*.

**Methods:**

Parasites infecting the body cavity of *Caiman yacare* in Pantanal wetlands, Mato Grosso do Sul, Brazil, were collected and processed for morphological studies using light and scanning electron microscopy. Genetic characterization was based on partial sequences of 18S and 28S rDNA. A phylogeny was reconstructed based on 28S sequences including all *Oswaldofilaria* available.

**Results:**

Nematodes were assigned to *O*. *bacillaris* mainly based on the oral opening markedly displaced ventrally. Males had seven pairs of caudal papillae encircling the cloaca, and their two most posterior pairs were hardly visible, which is also characteristic in other congeners. Moreover, a delicate area rugosa previously unreported in the species was observed. *Oswaldofilaria bacillaris* formed a monophyletic assemblage with the congeners, being sister to *O*. *chabaudi*, and *O*. *petersi* was basal, these last two parasites of lizards.

**Conclusion:**

The present findings strengthened the specific diagnosis of *O*. *bacillaris* and elucidated the morphological contradictions. The phylogeny reinforced the hypotheses that *Oswaldofilaria* emerged in lizards and colonized crocodilians by host switch, and reduction in number of caudal papillae in males is a derived feature.

## Introduction

Onchocercidae Leiper, 1911 (Spiruromorpha: Filairoidea) is a diverse family of nematodes with complex life cycles, in which the transmission is mediated by hematophagous arthropods [1, 2]. The microfilaria of onchocercids, a larval stage prior to L1, is the key element for their transmission and has been reported in all organ systems as well as in most tissues of their definitive hosts, although the preferred site varies according to the species [1]. Onchocercids infect a wide spectrum of hosts, including amphibians, reptiles, birds and mammals [2, 3]. Within Onchocercidae, the subfamily Oswaldofilariinae Chabaud & Choquet, 1953 incudes 7 genera of parasites mostly in lacertilian reptiles, except for the genus *Oswaldofilaria* Travassos, 1933 that has been reported also in crocodilians [4–6].

*Oswaldofilaria* is among the most diverse genera of onchocercids and currently comprises 15 valid species, which occur in Australia, Africa and South America, representing a “gondwanian-type” distribution [4, 6]. The following congeners have been reported in crocodilians: *O*. *bacillaris* (Molin, 1858), *O*. *kanbaya* Manzanell, 1986, *O. medemi* Marinkelle, 1981 and *O. vesterae* Bain, Kouyaté & Baker, 1982 [4, 7–10]. *Oswaldofilaria bacillaris* and *O*. *medemi* are the only species known to infect crocodilians in South America until now [4].

*Oswaldofilaria bacillaris* (= *Filaria bacillaris*) was originally described parasitizing *Melanosuchus niger* (Spix, 1825) and *Caiman crocodilus* (Linnaeus, 1758) from Brazil [7]. The morphology of this species has been investigated since then, but some morphological aspects remain poorly known and seemingly variable [11, 12]. This variability could be better understood with the help of genetic data, but there is no such data available for *O*. *bacillaris*.

The lack of genetic data is also observed in most species of *Oswaldofilaria*, in which only two of the 15 species have been genetically characterized, in addition to a few sequences not identified to species level. This panorama makes it difficult to understand the phylogenetic relationships among *Oswaldofilaria* spp., since some of their morphological features are reduced and seemingly show evolutionary convergence [3, 13, 14].

During parasitological analysis of a *Caiman yacare* (Daudin, 1801) (Crocodylia: Alligatoridae) found freshly dead at one lagoon of the Pantanal wetlands, State of Mato Grosso do Sul, Brazil, some specimens of *O*. *bacillaris* were found infecting its body cavity. The morphology of these parasites was evaluated in detail using light and scanning electron microscopy, and genetic characterisation based on two nuclear ribosomal markers was performed. The results are presented herein.

## Materials and Methods

### Collection, Processing and Morphological Evaluation of Parasites

On 18 August 2018 one specimen of *C*. *yacare* (about 1.5 m of total length) was found freshly dead by researchers of the Universidade Federal de Mato Grosso do Sul, at a marginal lagoon of the federal highway BR 262, which crosses Pantanal wetlands, municipality of Corumbá, State of Mato Grosso do Sul, Brazil. Host nomenclature and classification are according to [15]. The reptile was immediately necropsied in the field and the filariae were found still alive in the body cavity. The nematodes were washed in saline, fixed in hot 4% formalin and preserved in 70% ethanol. The middle body part of one male specimen was excised and fixed in molecular-grade 96–99% ethanol for genetic studies.

For morphological examination using light microscopy, specimens were cleared in glycerine and observed in a Nikon Eclipse Ei, with a PrimeCam Intervision 12 attached, which was used for measuring and image capture. Drawings were made in a microscope Olympus CH2 with a drawing tube attached. For scanning electron microscopy (SEM) one male and one female were dehydrated through a graded ethanol series, dried by evaporation with hexamethyl disilazane, coated with gold and observed in a JEOL JSM 6460-LV, at an accelerating voltage of 15 kV. Measurements are presented as ranges and in micrometres, unless indicated otherwise. Voucher specimens were deposited in the Coleção Helmintológica do Instituto Oswaldo Cruz (acronym CHIOC; accession number 39858).

The uterus of one gravid female was dissected and the microfilariae extracted, mounted in temporary slides with glycerine and observed using light microscopy.

## Genetic Procedures

Genomic DNA was isolated using DNeasy Blood & Tissue Kit (QIAGEN, Hilden, Germany), following the manufacturer’s instructions. Two regions of the nuclear rDNA were amplified: 18S using the primers Nema18SF (5′-CGCGAATRGCTCATTACAACAGC-3′) and Nema18SR (5′-GGGCGGTATCTGATCGCC-3′) [16], and 28S using the primers D2A (5′-ACAAGTACCGTGAGG GAA AGT-3′) and D3B (5′-TGC GAAGGAACCAGCTACTA-3′) [17]. The polymerase chain reactions (PCR) and cycling conditions were the same as those used by Ailán-Choke et al. [18]. An enzymatic treatment with ExoSAP-IT (ThermoFisher, Waltham, MA, USA) was carried out to purify the PCR amplicons that were sent for sequencing at ACTGene, Ludwig Biotec, Alvorada (Brazil).

Contiguous sequences were assembled and inspected, primers were trimmed, and the consensus was extracted in Geneious Prime (Dotmatics, Boston, MA, USA) and deposited in GenBank. A preliminary BLAST search on the GenBank database (https://www.ncbi.nlm.nih.gov/Blast.cgi) (accessed in December 2025) was performed to confirm the genetic proximity between the present samples and those from other species of *Oswaldofilaria*.

## Phylogenetic Analysis

Only the 28S sequences were used for phylogenetic reconstruction due to data availability and the genetic regions that were sequenced. These sequences were from representatives of the clade ONC 1, *sensu* Lefoulon et al. [3], which includes *Oswaldofilaria* and its closely related genera *Icosiella* Seurat, 1917 and *Ochoterenella* Caballero, 1944; the outgroup was chosen also according to Lefoulon et al. [3]. Information on the sequences used in the phylogenetic analysis can be found in Table 1. Sequences were aligned using M-Coffee [19] and evaluated by the transitive consistency score, to verify the reliability of aligned positions and trim ambiguous sites [20].

**Table 1:** Onchocercid taxa whose sequences were retrieved from GenBank and used in the phylogenetic analysis associated with host, accession number and geographic origin

Species	Host	GenBank	Geographic origin
*Filaria latala* (outgroup)	*Panthera leo*	KP760377	South Africa
*Oswaldofilaria chabaudi*	*Tropidurus torquatus*	KP760402	Brazil
*Oswaldofilaria petersi*	*Crocodilurus amazonicus*	KP760403	Peru
*Ochoterenella* sp. 1a	*Agalychnis callidryas*	MT153694	Costa Rica
*Ochoterenella* sp. 1b	*Rhinella granulosa*	KP760394	Venezuela
*Ochoterenella* sp. 2	*Rhinella marina*	KP760395	Venezuela
*Ochoterenella* sp. 3	*Phyllomedusa bicolor*	KP760393	French Guiana
*Icosiella neglecta*	*Pelophylax ridibundus*	OL351845	Albania

The phylogeny was reconstructed in BEAST 2 software using Bayesian Inference [21]. The best-fit substitution model for the 28S alignment was chosen according to bModelTest package implemented in BEAST 2 [22]; and the molecular clock model was relaxed optimized defined using the nested sampling method [23]. Parameter densities, ESS, burn-in, and the chain convergence were examined in Tracer [24].

The posterior estimates of parameter densities, the ESS for each parameter, and the posterior probability for nodal supports in the majority rule consensus phylogenetic tree were determined after running the Markov chain Monte Carlo (MCMC), using 4 chains in 2 runs for 10 × 10^6^ generations, with the sampling frequency at every thousand generation, with 25% burn-in, and saving the last 75% of generated trees.

## Results

A total of eight specimens of *O*. *bacillaris* were found in the single *C*. *yacare* examined, of which five were males and three were females. One male specimen was used for genetic characterisation (middle body part) and SEM observations (anterior and posterior ends). The following results are presented in two sections: one regarding the morphological description, and one regarding the genetic characterisation and phylogeny. Moreover, Table 2 contains the morphometric data of *O*. *bacillaris* from all taxonomic studies of the species in addition to the present one.

**Table 2:** Comparative measurements of *Oswaldofilaria bacillaris* from different taxonomic studies. Measurements are in micrometers unless otherwise indicated

	[11]	[12]	Present study
Host	Caiman crocodilus	Caiman crocodilus	Caiman yacare
Locality	Rio de Janeiro, Brazil	Belém, Brazil	Mato Grosso do Sul, Brazil
MALE	n = ?	*n* = 1	*n* = 3
Length (mm)	20	31.8	22.4–28.7
Width (mm)	210	310	201–251
Buccal capsule Length	16	10	29–40
Buccal capsule width	21	NM	40–64
Muscular oesophagus length	700*	575	366–726
Glandular oesophagus length (mm)	4.6	3.0*	3.4–4.3
Muscular glandular ratio	1 : 6.6	1 : 5.2*	1 : 5.8–7.5
Oesophagus total (mm)	5.3	3.6	3.7–5.0
Oesophagus ratio (%)	26.5*	11.3*	14–21
Nerve ring to anterior end	360	525	286–687
Deirids to anterior end	NM	875	576–817
Tail	140	265	162–242
Right spicule	180	160	143–162
Left spicule	380	450	367–403
Left to right spicule ratio	1 : 2.1*	1 : 2.8*	1 : 2.4–2.8
FEMALE	n = ?	*n* = 1	*n* = 4
Length (mm)	45	64	46.9–58.1
Width (mm)	250	375	261–357
Buccal capsule length	NM	NM	35–43
Buccal capsule width	NM	NM	56
Muscular oesophagus length	1,000*	NM	720–792
Glandular oesophagus length (mm)	8	NM	4.3–4.7
Muscular glandular ratio	1 : 8.0*	NM	1 : 5.4–6.5
Oesophagus total (mm)	9.0	4.8	5.1–5.4
Oesophagus ratio (%)	20*	7.5	9–11
Nerve ring to anterior end	600	NM	717–734
Deirids to anterior end (mm)	NM	1.25	1.1–1.2
Tail	NM	435	312–388
Vulva to anterior end (mm)	13–21	12.8	15.5
Vulva ratio (%)	NM	20*	32–34
Microfilariae length	NM	84–117	110–125
Microfilariae width	NM	6–9	6–12

*Measurements not given in the original study, but possible to estimate here. NM ,  not measured and impossible to estimate here

### Description of *Oswaldofilaria bacillaris* from *Caiman yacare*

Family Onchocercidae Leiper, 1911.

Subfamily Oswaldofilariinae Chabaud & Choquet, 1953.

Genus *Oswaldofilaria* Travassos, 1933.

Sepecies *Oswaldofilaria bacillaris* (Molin, 1858).

**Fig. 1:**
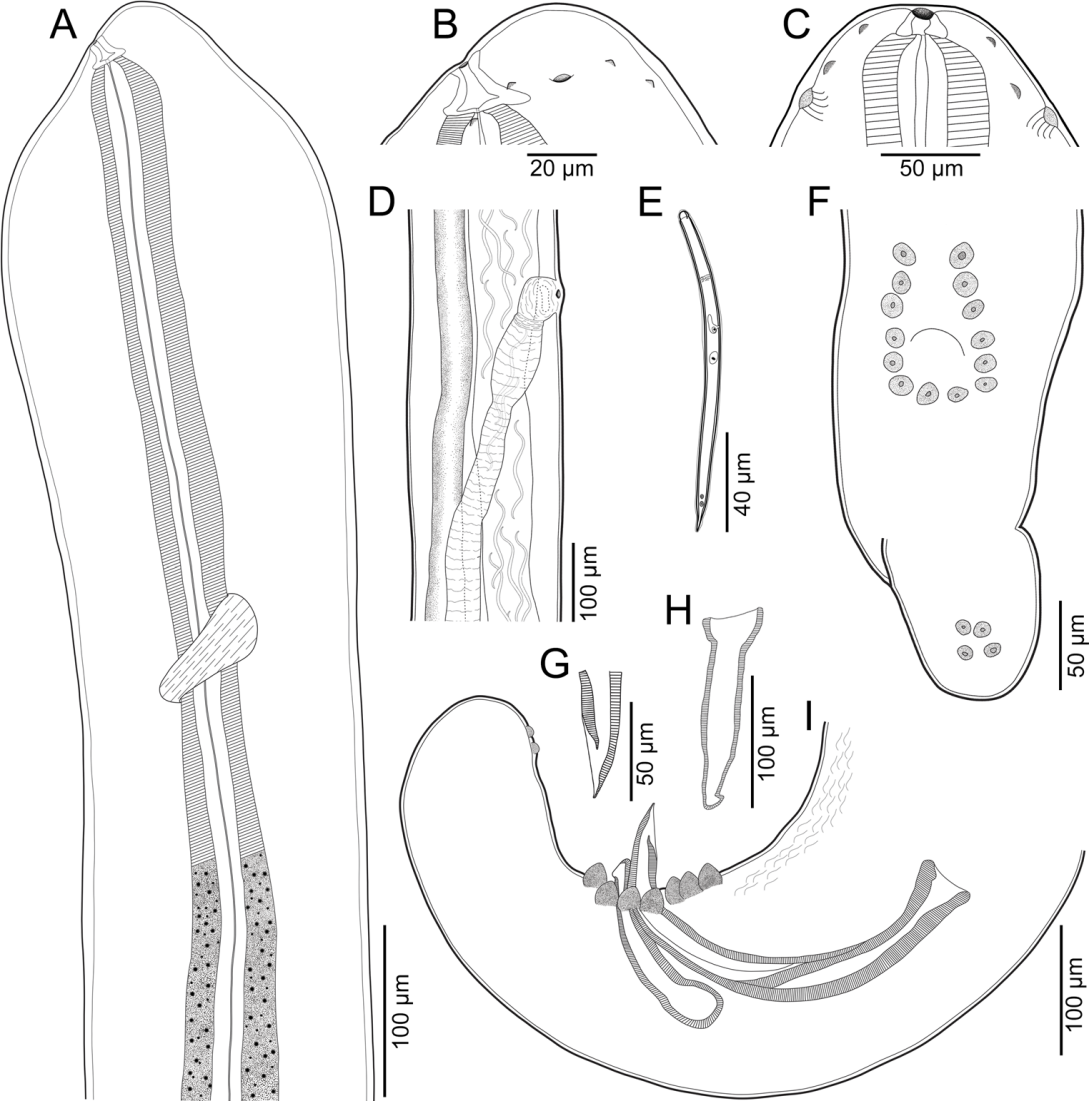
Line drawings of *Oswaldofilaria bacillaris* ex. *Caiman yacare*. **A**, anterior end of female, lateral view; **B**, cephalic end of female, lateral view; **C**, cephalic end of male, dorso-ventral view; **D**, region of vulva, lateral view; **E**, microfilaria extracted from uterus, lateral view; **F**, posterior end of male, ventral view; **G**, tip of left spicule, lateral view; **H**, right spicule, lateral view; **I**, posterior end of male, lateral view

**Fig. 2:**
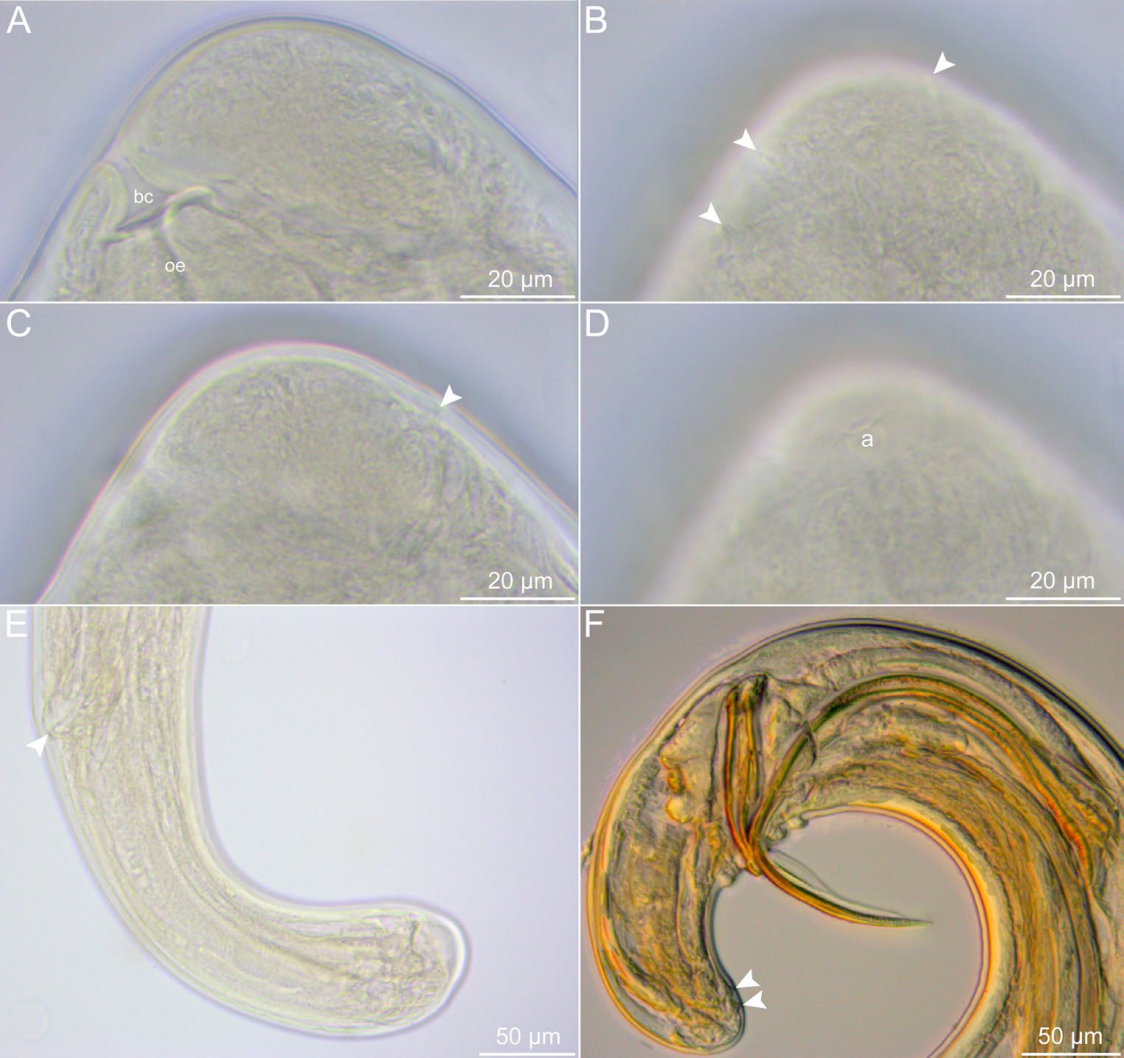
Light micrographs of *Oswaldofilaria bacillaris* ex. *Caiman yacare*. **A**–**D**, cephalic end of female, lateral views (arrowheads indicate the minute cephalic papillae); **E**, posterior end of female, lateral view (arrowhead indicates the anal opening); **F**, posterior end of male, lateral view (arrowheads indicate the inconspicuous most posterior caudal papillae). Abbreviations: a, amphid; bc, buccal capsule; oe, oesophagus

**Fig. 3:**
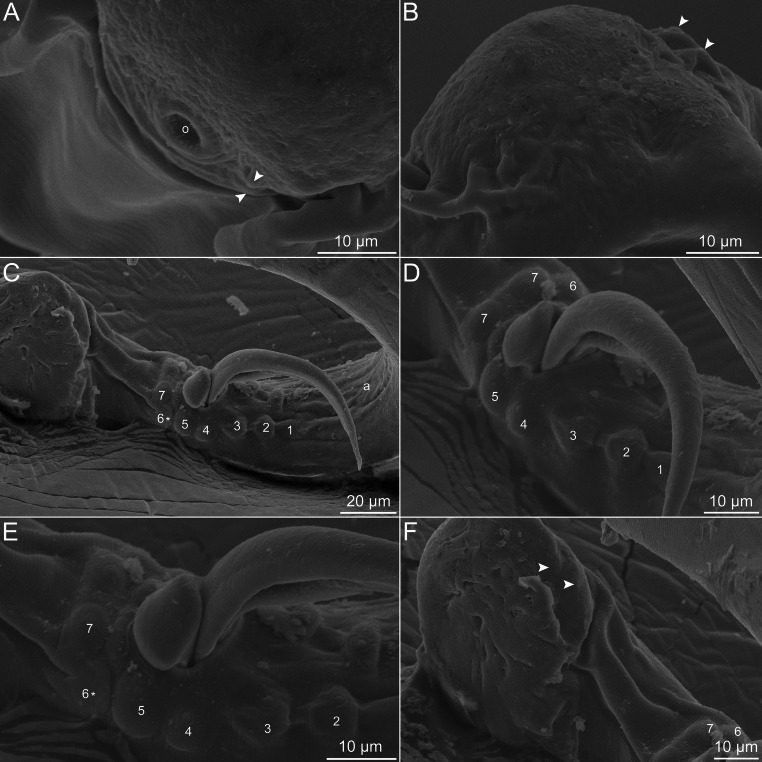
Scanning electron micrographs of *Oswaldofilaria bacillaris* ex. *Caiman yacare*. **A**, **B**, posterior end of female, subapical view and dorsal views, respectively (arrowheads indicate the minute cephalic papillae), **C**–**F**, Posterior end of male, subventral views (numbers indicate the caudal papillae of each pair that surround cloaca, in crescent order from the most anterior to the most posterior; asterisk indicate the damaged 6 papilla from left side; arrowheads indicate the most posterior inconspicuous papillae near tail tip). Abbreviations: o, oral opening

*General*: Long and thin nematodes, with typical filariform aspect. Opaque whitish colour when fresh and fixed. Cuticle delicate and smooth, lateral alae absent. Cephalic end narrower than body, dome-shaped, bearing two circles of minute (almost inconspicuous) cephalic papillae, one internal and one external, each circle with four papillae (Figs. 1A–C, 2A–D and 3A and B). Amphids with conspicuous opening, laterally displaced (Figs. 1B and C and 2D). Oral opening circular, small and ventrally directed (Figs. 1A–C, 2A and 3A). Buccal capsule present, cup-shaped with thick sclerotised wall (Figs. 1A–C and 2A). Long and slender oesophagus divided into anterior shorter muscular portion and much longer posterior glandular portion (Fig. 1A). Nerve ring encircling muscular oesophagus on its posterior third (Fig. 1A). Deirids small and slightly salient, button-shaped and located posterior to muscular-glandular oesophagus junction. Excretory pore not visualized. Sexual dimorphism evident. Males markedly smaller and thinner than females, with strongly coiled posterior end. Tail blunt in both male and female (Figs. 1F and I, 2E and F and 3C and F). Phasmids indistinct.

*Male (based on 4 adult specimens)*: Body 22.4–28.7 mm long, 201–205 wide at middle of body. Buccal capsule 29–40 long, 40–64 wide. Total length of oesophagus 3.7–5.0 mm, muscular and glandular portions 366–726 and 3.4–4.3 mm long, respectively. Oesophagus representing 14–21% of body length; muscular to glandular oesophagus ratio 1:5.8–7.5. Nerve ring and deirids located at 286–687 and 576–817 from anterior end, respectively. Posterior end strongly coiled. Ventral delicate area rugosa present, almost indistinct, with short extension, ending slightly anterior to fist pair of caudal papillae and extending anteriorly about 643–795 (Figs. 1G and 3C). Fourteen caudal papillae arranged in pairs, forming U shape around cloaca (Figs. 1F and I, 2F and 3C–E), plus two pairs of ventral inconspicuous papillae, slightly anterior to tail end (Figs. 1F and I, 2F and 3F). Spicules robust, different in shape and length (Figs. 1I and 2F and C–E). Smaller spicule (right) 143–162 long, with cup-shaped proximal end and barb at distal end (Figs. 1H and I, 2F and 3C–E). Longer spicule (left) sheathed, 367–403 long, spear-shaped, with expanded and straight proximal end and pointed distal end, shaft located somewhat at its mid length (Figs. 1G and I, 2F and 3C–E). Gubernaculum absent. Tail blunt, 643–795 long.

*Female (based on 3 specimens with microfilariae in uterus)*: Body 46.9–58.1 mm long, 261–357 wide at middle of body. Buccal capsule 35–43 long, 56 wide. Total length of oesophagus 5.1–5.4 mm, muscular and glandular portions 720–792 and 4.3–4.7 mm long, respectively. Oesophagus representing 9–11% of body length; muscular to glandular oesophagus ratio 1:5.4–6.5. Nerve ring and deirids located at 714–734 and 1.1–1.2 mm from anterior end, respectively. Vulva pre-equatorial, 15.5–19.1 mm from anterior end, at about 32–34% of body length. Vagina subspherical and muscular, posteriorly directed and separated from ovijector by muscular ring sphincter (Fig. 1D). Ovijector long and weakly muscular, its distal end expanded, forming barrel-shaped chamber (Fig. 1D). Didelphic and amphidelphic uterus, full of microfilariae. Tail long, directed dorsally, with rounded end, and without projections, 312–338 long (Fig. 2E).

*Microfilaria (based on 8 specimens*,* extracted from the uterus of one female)*: Body 110–125 long and 6–12 wide. Tightly sheathed, elongated and slender, with notch at cephalic region, conical posterior end, and pointed tip; cephalic hook ventrally displaced, primordial nerve ring, excretory cell and pore, and G cell visible in some specimens, at about 30, 40 and 60 from anterior end, respectively; two posterior nuclei visible at tail region (Fig. 1E).

## Genetic Characterisation and Phylogeny

We obtained partial fragments of the 18S (866 bp) and 28S (788 bp) rDNA for *O*. *bacillaris* (GenBank accession numbers PZ058501, PZ058504). The BLAST search indicated that the present sequences were most similar to those of other species of *Oswaldofilaria*, in which the 18S sequence was 99.8% similar to that of *O*. *chabaudi* Pereira, Souza Lima & Bain, 2010 (LR594609), and the 28S sequence was 92.2% and 91.7% similar to those of *O*. *chabaudi* (KP760402) and *O*. *petersi* Bain & Sulahian, 1974 (KP760403), respectively.

**Fig. 4:**
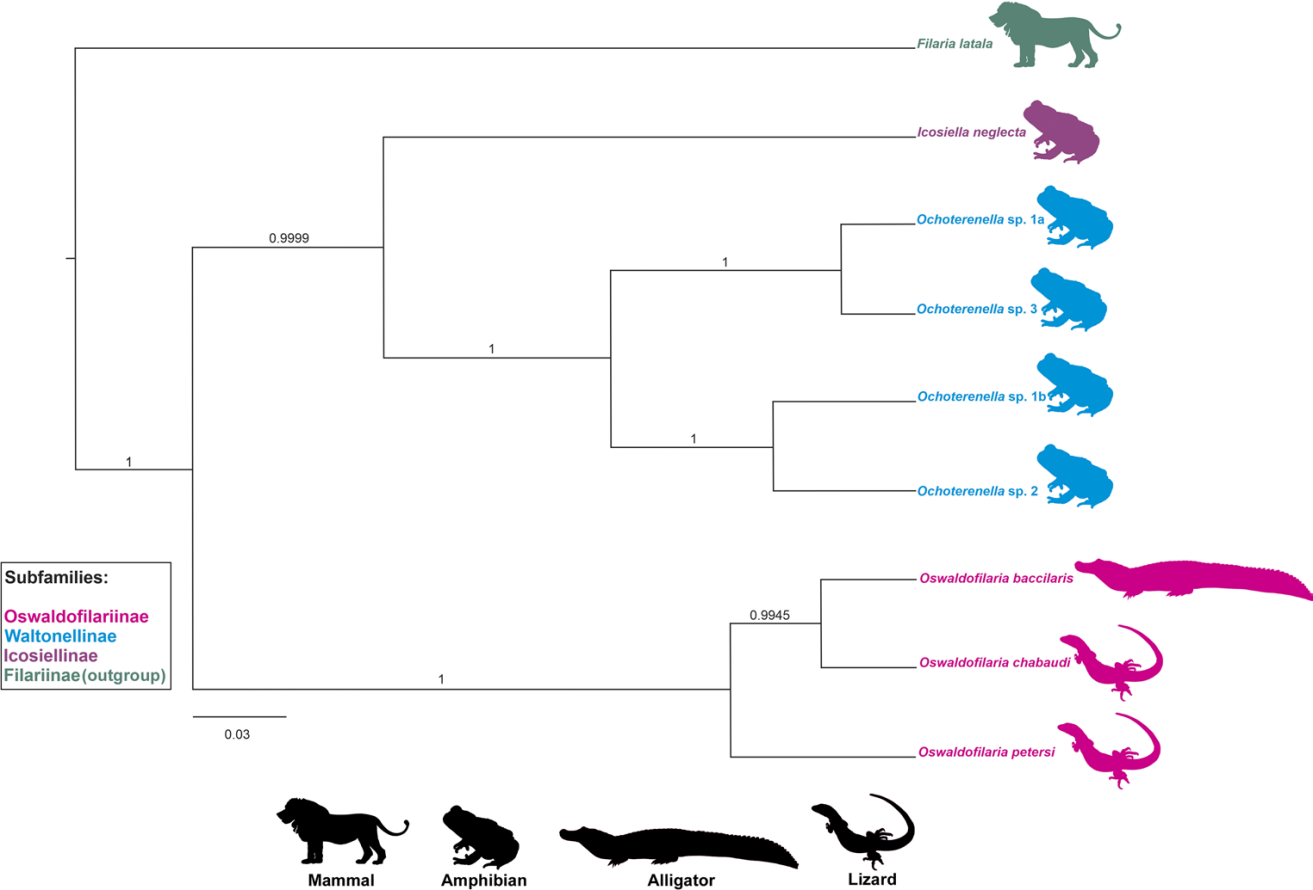
Phylogenetic reconstruction based on Bayesian inference, from 28S rDNA sequences of onchocercids from clade ONC1 (*sensu* [3]) that includes *Oswaldofilaria* and closely related genera. Numbers indicate branch supports given as Bayesian posterior probabilities, after running the Markov Chain Monte Carlo, in duplicate for 10 × 10^6^ generations, 1% sample frequency and 25% burn-in. Scale bar indicates number of substitutions per site

*Oswaldofilaria bacillaris* grouped with the other two congeners that have 28S sequences available in GenBank, in which the species was sister to *O*. *chabaudi* and both were siter to *O*. *pertersi* (Fig. 4). Oswaldofilariiane was sister to a clade composed of Icosiellinae represented by one genetic sequence, and of Waltonellinae that was monophyletic (Fig. 4). All branch supports were high in the phylogenetic reconstruction from 28S sequences (Fig. 4).

## Discussion

The original description of *O*. *bacillaris* was poorly detailed and brief regarding the morphological aspects of the species [7]. Subsequently, two studies assessed the morphology of *O*. *bacillaris* in more detail [11, 12]. However, Travassos [11] reported only 7 pairs of caudal papillae in males, whereas Prod’hon and Bain [12] reported 2 additional pairs near the tail end and an unpaired papilla that was mentioned in the text, but not depicted it in the line drawings. These morphological contradictions will be discussed in relation to what was observed in the present material as follows.

The present material showed a morphology that resembled most the description of *O*. *bacillaris* by Prod’hon and Bain [12], with differences regarding the supposed unpaired papilla and the lack of area rugosa in males, as well as the nature of the sheath surrounding the microfilaria. Probably, the unpaired papilla reported by these authors may have been a textual error, since it does not appear in their line drawings. The absence of area rugosa may be related to the delicate nature of this structure, which is hardly visible using light microscopy - the only method that was used by Prod’hon and Bain [12]. The loosened sheath of microfilariae mentioned by Prod’hon and Bain [12] in contrast to the tight one observed in the present specimens, may be related to their development. Prod’hon and Bain [12] obtained fully developed microfilariae in experimental infections, whereas here we obtained them directly from the uterus, mostly in early stages of development. This may be also the reason for the presence of a cephalic notch that was observed only in the larvae obtained here.

As same as the area rugosa, the two pairs of caudal papillae near the tail end of males of *O*. *bacillaris* were hardly visible in both light and scanning electron microscopy. In fact, it was easier to observe their internal structures under light microscopy. This may have been the reason why Travassos [11] did not observe them.

The morphometry of *O*. *bacillaris* has been little documented, the existing data are limited to measurements of some characters of a few specimens [11, 12; see also Table 1], which makes comparisons based on such data difficult. In this sense, the present results that included more detailed information on the morphometry of *O*. *bacillaris*, can be important for future studies evaluating intraspecific variations. Nevertheless, the morphometry of the newly collected material was mostly within the range reported in previous descriptions of *O*. *bacillaris*, thus showing no substantial differences [11, 12; see also Table 1]. The small differences might be population variations.

With the further description of *O*. *bacillaris* provided in the present work, including the first observation of the species using SEM, its specific diagnosis could be strengthened. Based on our results it was possible to resolve the inconsistencies between the previous taxonomic descriptions [i.e., 11, 12], as well as observe for the first time the delicate area rugosa in males, which is characteristic of several *Oswaldofilaria* spp.

*Oswaldofilaria bacillaris* was originally described by Molin [7] as *F*. *bacillaris*, and with the erection of *Oswaldofilaria* by Travassos [11], it was transferred to this genus as the type species. Since then, 14 congeners have been described, three of which are parasites in crocodilians [4, 5]. *Oswaldofilaria bacillaris* remains the only species of the genus easily recognizable by its characteristic cephalic end, with the oral opening markedly displaced ventrally and the well-developed amphids (see also [12]).

Interestingly, the two terminal pairs of caudal papillae in males of *Oswaldofilaria* spp. seem to be consistent and, in some species, for example, *O*. *azevedoi* Bain, 1974, *O*. *belemensis* Bain & Sulahian, 1974, *O*. *bacillaris*, *O*. *brevicaudata* (Rodhain & Vuylsteke, 1937) and *O*. *versterae*, they are reduced or inconspicuous [10, 12, 25–27]. Therefore, in the few congeners in which this character is not mentioned, such as *O*. *medemi* Marinkelle, 1981, there is a possibility that it was overlooked. In this sense, when using the caudal papillae of males as diagnostic features, we recommend relying on those papillae that are not the terminal ones. Regarding the caudal papillae disposed around the cloacal opening, *O*. *bacillaris* resembles *O*. *medemi* and *O*. *versterae*, parasites of crocodilians from South America and South Africa, respectively. However, males of *O*. *medemi* have six pairs of caudal papillae instead of seven as in those of *O*. *bacillaris* [9], and this last differs from *O*. *medemi*, *O*. *versterae* and from all other congeners based on the oral opening ventrally displaced, as previously mentioned.

There are two evolutionary hypotheses for *Oswaldofilaria*, one posits that the ancestral and the early species were parasites of lizards, later colonizing and deriving in crocodilians by host switch [10]. On the contrary, the other hypothesis asserts that the ancestral was a parasite of crocodilians, which was supported by the old idea that crocodilians were more ancestral than lizards [10]. In this sense, Bain et al. [10] considered the following characters that are consistently present in species of *Oswaldofilaria* parasitic in crocodilians as ancestral: reduced number of caudal papillae in males (ten or less pairs) arranged in a strongly bisymmetric pattern, long oesophagus in both male and female, and very unequal spicules in that the smaller represents less than a half length of the longer.

The phylogenetic results, however, supported the host switch hypothesis, since *O*. *bacillaris* was sisters to *O*. *chabaudi*, which is a parasite of lizard, and *O*. *petersi* that is also a parasite of lizard, was basal within the clade of *Oswaldofilaria*. In addition, such a phylogenetic pattern seems to agree with another hypothesis on Onchocercidae, that the reduction in number of caudal papillae and their distribution surrounding the cloacal opening (as in *O*. *bacillaris* and *O*. *chabaudi*) is derived, in relation to numerous papillae distributed in rows along the tail of males (as in *O*. *petersi*) [3, 28, 29]. However, it should be mentioned that the results are preliminary, since only three species of *Oswaldofilaria*, out of 15, have been genetically characterised.

This first genetic characterization of *O*. *bacillaris* and the phylogenetic analysis confirmed its status as type species of the genus, agreeing with the proposition of Travassos [11]. Moreover, the results, although preliminary, provided interesting insights into the evolution of *Oswaldofilaria* spp. and could confirm some morphological hypotheses on Onchocercids. The detailed morphological evaluation of the species revealed a previously unreported feature (area rugosa) and could clarify the associated inconsistencies. This is the first report of *O*. *bacillaris* infecting *C*. *yacare* as well as in the Pantanal wetlands biome.

## Data Availability

All data is available in the manuscript or deposited in public repositories (biological collection and GenBank).
